# Probiotic Bacteria in Stimulating Human Physiological Responses: Metabolic Function and Overall Health

**DOI:** 10.3390/foods15122079

**Published:** 2026-06-08

**Authors:** Shin-Yee Chong, Raja Balqis Raja-Razali, Nor Hidayah Ismail, Muhammad Ameer Ushidee-Radzi, Nur Asyiqin Zahia-Azizan, Muthukumaaran Manickam, Danial ‘Aizat Norhisham, Zul Ilham, Anna Reale, Stefania Nazzaro, Daniela Iovanna, Wan Abd Al Qadr Imad Wan-Mohtar, Tiziana Di Renzo

**Affiliations:** 1 Functional Omics and Bioprocess Development Laboratory, Institute of Biological Sciences, Faculty of Science, Universiti Malaya, Kuala Lumpur 50603, Malaysia; helenchong@um.edu.my (S.-Y.C.); rajablqslegit@gmail.com (R.B.R.-R.); ameerushidee@um.edu.my (M.A.U.-R.); asyiqinazizan@um.edu.my (N.A.Z.-A.); muthukumaranmuruga@gmail.com (M.M.);; 2 Centre for Research in Biotechnology for Agriculture (CEBAR), Universiti Malaya, Kuala Lumpur 50603, Malaysia; norhidayah@um.edu.my; 3 Biomass Energy Laboratory, Faculty of Science, University Malaya, Kuala Lumpur 50603, Malaysia; ilham@um.edu.my; 4 Centre for Science and Environment Studies, Institute of Islamic Understanding Malaysia, 2 Langgak Tunku Off Jalan Tuanku Abdul Halim, Kuala Lumpur 50480, Malaysia; 5 Institute of Food Sciences, National Research Council (CNR-ISA), Via Roma, 64, 83100 Avellino, Italy; anna.reale@isa.cnr.it (A.R.); stefania.nazzaro@isa.cnr.it (S.N.); danielaiovanna@cnr.it (D.I.); 6 Center of Excellence for Research, Value Innovation and Entrepreneurship (CERVIE), UCSI University, Cheras, Kuala Lumpur 56000, Malaysia

**Keywords:** microbiota, gut–brain axis, psychobiotics, immune system, functional foods, precision nutrition

## Abstract

This review examines the functional role of lactic acid bacteria (LAB) and Bifidobacteria in modulating host physiology through interconnected metabolic, immune, and neuroendocrine pathways. These effects are particularly relevant in gastrointestinal diseases, where dysbiosis is associated not only with local intestinal dysfunction but also with systemic conditions, including metabolic syndrome, infections and complications in immunocompromised individuals. LAB and Bifidobacteria play key roles through the production of short-chain fatty acids, which contribute to maintaining intestinal barrier integrity, regulating lipid and glucose metabolism, improving insulin sensitivity, and exerting anti-inflammatory effects that may reduce the risk of metabolic disorders. Beyond metabolic regulation, the review explores the emerging concept of psychobiotics, focusing on how probiotic bacteria modulate host physiology through integrated metabolic, immune and neuroendocrine mechanisms. Current evidence suggests that these effects are highly strain-specific and influenced by dosage and study design, and host-related factors, often arising indirectly through complex host-microbe interactions rather than direct neurotransmitter activity. Although increasing evidence links these microorganisms to stress regulation, mood, and cognitive function, findings from human studies remain inconsistent. Therefore, well-designed clinical trials, combined with multi-omics approaches, are needed to clarify underlying mechanisms and substantiate clinical efficacy. Overall, probiotics-based strategies offer a promising and practical approach to supporting metabolic health and general psychological well-being through functional foods.

## 1. Introduction

Probiotics are defined as “live microorganisms that, when administered in adequate amounts, confer health benefits on the host” [1,2]. Hill et al. [3] established that probiotics must have “defined contents, an adequate number of viable organisms at the end of the shelf life, adequate evidence of health benefits,” and that all probiotics must be “safe for their intended use.” This definition was then confirmed in 2017 by the International Scientific Association for Probiotics and Prebiotics (ISAPP) in an official statement [4,5]. The regulatory classification of probiotics, however, is not uniform and may vary depending on their intended use, formulation, route of administration, and associated health claims. According to the US Food and Drug Administration (FDA), probiotics may be regulated as dietary supplements, foods, medical foods, biological products, or pharmaceutical/drug products depending on the specific product category and health claims [6]. The mechanisms through which probiotics exert their effects are multifactorial. These include: (a) modulation of gut microbial balance, promoting the growth and predominance of beneficial bacteria [7]; (b) regulation of inflammatory responses; (c) enhancement of the host’s innate and adaptive immune systems [8,9]; (d) reinforcement of the intestinal immune barrier; and (e) production of antimicrobial and other bioactive compounds [9].

Different species of LAB (Lactobacillaceae and Streptococcaceae) and Bifidobacteriaceae are widely recognized for their probiotic potential. Representative examples include *Lactobacillus delbrueckii*, *Lactiplantibacillus plantarum*, *Lacticaseibacillus casei*, *Lactobacillus acidophilus*, *Limosilactobacillus reuteri*, *Streptococcus thermophilus*, *Bifidobacterium breve*, *Bifidobacterium longum*, and * Bifidobacterium bifidum* [10]. To a lesser extent, probiotic properties have also been reported for *Pediococcus acidilactici*, *Pediococcus pentosaceus*, and *Enterococcus* species. These microorganisms are widely incorporated into dietary supplements, fermented foods, and dairy products due to their established health benefits [11]. In this context, probiotics have been associated with the prevention and management of Non-communicable diseases (NCDs) and Major depressive disorder (MDD) [12,13,14,15]. NCDs are non-infectious conditions characterized by multiple risk factors, long latency periods, and chronic progression, often leading to functional impairment, disability, and limited curability [16]. The most prevalent NCDs include cardiovascular diseases, cancer, chronic respiratory diseases, diabetes mellitus, neurodegenerative disorders, chronic kidney disease, and autoimmune conditions [11,15,17,18]. In contrast, MDD is a psychiatric condition characterized by persistent depressed mood, sleep disturbances, cognitive impairment, and a range of emotional symptoms, including guilt, fatigue, and changes in appetite and body weight, along with an increased risk of suicide [19].

Within this framework, the gut–brain axis plays a central role as a bidirectional communication network linking the central nervous system (CNS) and the enteric nervous system (ENS). The gut is often referred to as the “second brain” due to its complex neural network and its close association with the gut microbiota. The conceptual relationship between the gastrointestinal system and the CNS has evolved considerably over time, culminating in the modern model of the microbiota–gut–brain axis (MGBA) [20]. Early concepts such as the “abdominal brain” and “nervous sympathy” were introduced to describe the close interconnection between digestive and nervous system functions [20,21,22]. Contemporary research has further demonstrated that the gut microbiota and the brain communicate through multiple interconnected pathways, including neural, endocrine, immune, and metabolic signaling mechanisms [23].

The consumption of probiotic-containing dairy products, such as yogurt and fermented milk, has been widely associated with a range of health benefits [15]. In this context, the traditional gut–brain axis has been further expanded into the food–gut–brain axis, emphasizing the role of diet as a key modulator of the gut microbiota and, consequently, brain function [15,24,25]. This emerging framework is supported by the complex enzymatic activity of LAB and Bifidobacteria in food systems, which facilitates the release of a wide range of bioactive compounds within the gut and the food matrix. These metabolites can influence brain function, metabolic processes, and systemic immune responses [26,27,28,29].

Together, the food–gut–health axis and the microbiota–gut–brain axis contributed to the emergence of “psychobiotics”, defined as probiotics capable of exerting beneficial effects on mental health [30]. A recent analysis by Global Market Insights [31] predicted that the psychobiotics market will expand from USD 353.8 million in 2025 to USD 936.8 million in 2034, with a compound annual growth rate (CAGR) of 11.4%. This growth reflects increasing scientific, clinical, and commercial interest in microbiome-based mental health interventions. Despite increasing evidence supporting the MGBA, the precise mechanisms through which probiotics influence metabolism and neurological functions remain only partially understood.

This review synthesizes current knowledge regarding the contribution of probiotics to metabolic and neurological regulation, with particular emphasis on their role in modulating host metabolic processes. It further evaluates existing evidence on strain-specific probiotic effects, underlying mechanistic pathways, and potential therapeutic applications.

A comprehensive literature search was conducted using the ISI Web of Science, Scopus, Google Scholar, and PubMed databases. The search strategy included the following descriptors: “probiotic bacteria” OR “psychobiotics” OR “lactic acid bacteria” OR “Bifidobacteria” AND (“metabolic regulation” OR “health” OR “functional foods” OR “gut–brain axis”). Publications published between 2006 and 2026 were considered. Only English-language articles focusing on probiotic-mediated metabolic regulation, gut microbiota modulation, functional food applications, and gut–brain axis interactions were included. Following the application of the inclusion criteria, titles and abstracts were screened, duplicate records were removed, and additional relevant studies were identified through the reference lists of the selected articles.

## 2. Taxonomy and Mechanistic Action of Lactic Acid Bacteria and Bifidobacteria

LAB are phylogenetically associated with the *Bacillota phylum* [32]. The principal genera with probiotic relevance include *Lactobacillus* (recently reclassified into several new genera), *Streptococcus*, and *Pediococcus* [10].

Species within the Lactobacillaceae family are microaerophilic, rod-shaped, lactic acid–producing bacteria that colonize the oral cavity, gastrointestinal tract, and vagina [33,34]. They ferment carbohydrates (glucose, lactose, sucrose), producing lactate, ethanol, and carbon dioxide. Based on metabolism, they are classified as homofermentative (e.g., *Lactobacillus delbrueckii*, *Lactobacillus helveticus*, *Lactobacillus crispatus*) or heterofermentative (e.g., *Levilactobacillus brevis*, *Limosilactobacillus fermentum*, *Lentilactobacillus buchneri*) [10,34]. Recent taxonomic revisions reassigned several species into new genera, including *Lactiplantibacillus plantarum*, *Lacticaseibacillus casei*, and *Lacticaseibacillus rhamnosus*. These bacteria produce bioactive compounds such as lactic acid isomers, vitamins, exopolysaccharides (EPS), bacteriocins, and antioxidants, contributing to oxidative stress modulation and cellular signaling, supporting their probiotic functionality [35,36,37,38].

*Pediococcus* comprises non-motile, coccoid LAB forming tetrads and showing mainly homofermentative metabolism. They ferment sugars into lactate via the Embden–Meyerhof–Parnas pathway, with some species shifting metabolism under aerobic conditions. They are classified mainly as *P. acidilactici* and *P. pentosaceus* [39,40]. Certain LAB enhance nutrient bioavailability by degrading complex molecules. Genera such as *Lactiplantibacillus*, *Lactococcus*, *Leuconostoc*, *Streptococcus*, and *Enterococcus* are important in fermentation, especially in dairy products [41].

Similarly, species of the genus *Bifidobacterium* are pleomorphic, anaerobic, acid-tolerant, rod-shaped microorganisms belonging to the Actinobacteria [32]. They commonly inhabit the human oral cavity, gastrointestinal tract, and vagina [42], and include clusters such as *B. bifidum*, *B. longum*, and *B. adolescentis*. They produce acetate via polysaccharide fermentation (fructose-6-phosphate pathway) [34], and some species are genetically adapted to encode glycosyl hydrolases, enabling the utilization of complex carbohydrates derived from both human and plant sources [43].

Many probiotic bacteria, especially members of *Lactobacillus* and *Bifidobacterium*, are recognized as safe, holding either “Generally Recognized as Safe” (GRAS) status or European Food Safety Authority Qualified Presumption of Safety (QPS) status. This supports their widespread application in functional foods and probiotic formulations. These microorganisms contribute to host health through nutrient metabolism, inhibition of pathogenic microbes, and modulation of inflammatory responses [44].

However, despite their generally favourable safety profile, certain species within the genera *Streptococcus* and *Enterococcus* may act as opportunistic pathogens, particularly in immunocompromised individuals [45]. Therefore, rigorous genomic and phenotypic characterization, including the assessment of antibiotic resistance, virulence factors, and strain-specific pathogenicity, remains essential prior to their application in food systems or probiotic products. Enterococci are gut commensals but can be opportunistic pathogens [45]. Some strains show probiotic traits (adhesion, bile and acid tolerance, enterocin production), but their use is limited due to safety concerns and lack of GRAS status; safety is strain-specific. Common strains include *Enterococcus faecium* SF68 and M74, used for diarrhea, irritable bowel syndrome, and intestinal disease prevention. Some strains also show antimutagenic, anticarcinogenic, cholesterol-lowering, and immunomodulatory effects, and may produce beneficial metabolites such as short-chain fatty acids. However, rigorous safety assessment remains essential.

### 2.1. Mechanisms of Colonization and Epithelial Adhesion

Different species belonging to the Lactobacillaceae and Enterococcaceae families (*Enterococcus* and *Tetragenococcus* genera), *Lactococcus* and *Bifidobacterium* genera, exert their beneficial effects primarily through colonization of the human gastrointestinal tract, particularly at the mucosal layer. Their ability to adhere to intestinal surfaces is largely attributed to a high affinity for host receptor sites, such as mucus-binding proteins (MUBs), which enables them to compete with and exclude pathogenic microorganisms [46,47].

MUBs are large, cell-wall-anchored proteins located on the surface of these bacteria. These proteins facilitate adhesion by interacting with mucins, the glycoprotein components that form the mucus layer lining the intestinal epithelium [48]. This mechanism has been well documented in species such as *Limosilactobacillus reuteri* and *L. fermentum* [49]. Lipoteichoic acid (LTA) is another important cell-surface component that mediates epithelial adhesion [46]. LTA is formed by linking teichoic acid (a major constituent of the Gram-positive wall) to lipids in the plasma membrane. Binding occurs through interaction between the LTA lipid moiety and epithelial receptors, such as adhesins or fibronectin [46]. Several probiotic species, such as *Lactobacillus johnsonii*, *L. buchneri*, and *B. bifidum*, use LTA as a mechanism for host attachment [50,51].

EPS play a multifunctional role in host–microbe interactions by modulating non-specific adhesion to epithelial surfaces and providing protection against antimicrobial compounds, bacteriophages, and phagocytosis [52]. Their effects on bacterial adhesion are highly context-dependent. EPS may enhance adhesion by increasing cell surface hydrophobicity, inhibit adhesion by masking receptor-binding sites, or modulate bacteria–mucin interactions under different gastrointestinal conditions, as observed in species such as *L. rhamnosus* and *P. pentosaceus* [53]. In addition, EPS can promote biofilm formation, which further supports bacterial persistence in the host environment.

Biofilm development provides increased resistance to harsh gastrointestinal conditions, including low pH, pancreatic enzymes, and bile salts, thereby enhancing colonization and survival [52,54,55]. The functional diversity of EPS is largely determined by their physicochemical properties, molecular structure, quantity, and composition [56].

Pili and flagella are surface structures that typically provide motility. While commonly associated with pathogenic bacteria such as *Escherichia coli* [46], they have also been identified in some LAB, including *Ligilactobacillus ruminis* and *Ligilactobacillus agilis* [57,58]. In these species, flagellin proteins appear to contribute more to immunomodulatory responses, such as interleukin secretion, rather than directly mediating adhesion. Meanwhile, several types of pili, like Type I and Type IV, contribute to adherence to host cells. Pilus-like structures have been found in several *Lactobacillus* and *Bifidobacterium* species, which enhance colonization and adhesion to mucosal surfaces [59]. For instance, *L. rhamnosus* produces SpaCBA pili, in which the SpaC subunit plays a key role in strengthening adhesion to gastrointestinal mucins. Similarly, several *Bifidobacterium* species possess genes encoding sortase-dependent pili and Type IV tight adherence pili (Tad), as observed in *B. breve* [60].

Adhesion to the intestinal surface can also be aided by bacterial proteinaceous S layer, the outermost component of the bacterial cell wall (40–200 kDa; 5–25 nm thick).

**Figure 1:**
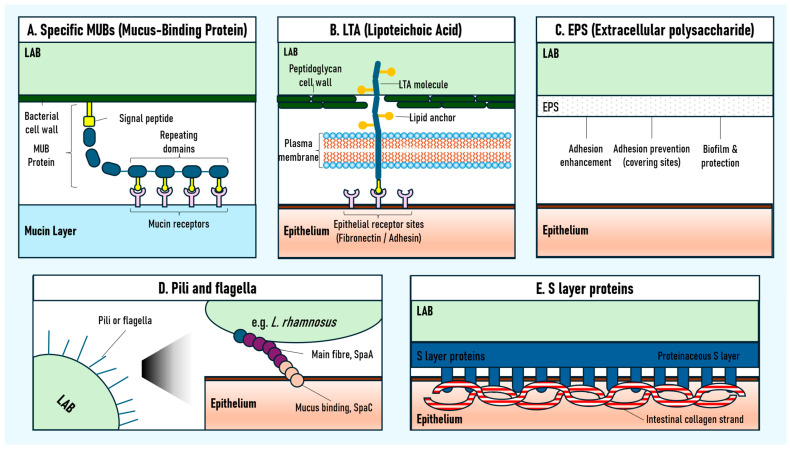
Illustrations of the mechanisms of adhesion and colonization of lactic acid bacteria in humans. (**A**) Direct binding to mucin glycopeptides via mucin-binding proteins (MBPs) [48]. (**B**) Binding to host cell surface receptors via lipoteichoic acid (LTA) [46]. (**C**) Non-specific, protective, and competitive binding via exopolysaccharides (EPS) [52,53,56]. (**D**) Enhanced colonization and adhesion via pili and flagella [59]. (**E**) Binding via the repetitive microstructures of S-layer proteins to intestinal collagen [62,64].

S-layer proteins, with their repeating microstructures, promote binding to intestinal epithelium collagen and can outcompete pathogens for attachment sites, thereby providing protection against infection [61]. Evidence of S-layer protein production was found in Lactobacilli such as *L. crispatus*, *L. brevis* and *L. helveticus*, particularly during the proliferation phase [62]. Klotz et al. [63] revealed that the deletion of the S-layer-related gene reduces the adhesive capacity of *L. acidophilus*, highlighting the importance of the structure in microbial attachment. Figure 1 shows a schematic representation of the different mechanisms of epithelial adhesion by LAB.

### 2.2. Production of Postbiotics: Short-Chain Fatty Acids and Bacteriocins

Postbiotics are defined as “preparation of inanimate microorganisms and/or their components that confers a health benefit on the host” [65]. They consist of inactivated microbial cells, cell fractions, and/or metabolites and cell-derived byproducts that are responsible for the observed biological effects [36,65].

Among the most important postbiotic compounds produced by LAB are short-chain fatty acids (SCFAs) and bacteriocins, which exhibit antimicrobial, immunomodulatory, and metabolic activities [66]. SCFAs are the principal end-products of anaerobic fermentation of dietary carbohydrates and fibers by the gut microbiota, specifically Proteobacteria and Actinobacteria in early life, Firmicutes and Bacteroidetes in adult ones and Proteobacteria in elderly ones (e.g., Enterobacteriaceae, Bifidobacteriaceae, Lactobacillaceae, Ruminococcaceae and Lachnospiraceae). Furthermore, various authors report that strains belonging to the genera *Eubacterium*, *Roseburia*, and *Faecalibacterium* are among the primary producers of SCFAs, which are beneficial to human health [67,68]. The most abundant SCFAs are acetate, propionate, and butyrate [69]. These metabolites play essential roles in maintaining intestinal barrier integrity, modulating host immune responses, and supporting neuronal function [70,71], and serving as key energy substrates for colonocytes [72]. Recent studies indicate that members of the genus Lactobacillaceae family are among the most prolific producers of postbiotic metabolites. For example, *L. plantarum* and *L. rhamnosus* can generate substantial amounts of butyrate and propionate when co-fermented with prebiotic substrates [73]. Similarly, *L. acidophilus* produces a broad spectrum of SCFAs, including propionic, isobutyric, butyric, and valeric acids [74], while *L. fermentum* has been associated with the production of isovaleric acid and propionate. Isovaleric acid and propionate were, instead, linked to *L. fermentum* [75]. Within the *Bifidobacterium* genus, *B. longum* and *B. bifidum* are key producers of acetate and propionate, respectively [75], while *Bifidobacterium animalis* generates both lactic and acetic acids [66]. Metabolically, SCFAs influence host physiology by binding to free fatty acid receptors FFAR2/FFAR3 on enteroendocrine and enterochromaffin cells. This interaction stimulates the release of hormones such as glucagon-like peptide-1 (GLP-1), peptide YY, as well as neurotransmitters such as serotonin. Collectively, these mediators regulate insulin secretion, appetite control, and CNS function via endocrine and vagal pathways [76,77].

From a neurological perspective, SCFAs affect the gut–brain axis through neural, endocrine, immune, and humoral pathways. They can influence neurotransmitter availability, regulate neuroinflammatory processes, and contribute to microglial maturation, thereby potentially affecting behavioural outcomes such as anxiety and depression [78,79]. However, direct causal evidence in humans linking specific SCFAs profiles to anxiety or depressive disorders remains limited, highlighting the need for further large-scale clinical investigations [79]. Bacteriocins, instead, are antimicrobial compounds produced by probiotic bacteria to inhibit competing microorganisms [80,81]. They are typically classified into three main groups based on their structure and post-translational modifications: lantibiotics (class I), small non-modified peptides (class II), and large heat-labile proteins (class III). At the genetic level, bacteriocin production is organized in operon-like clusters that include structural genes, immunity proteins, secretion systems, and regulatory elements, enabling coordinated synthesis and self-protection [80,81]. Prominent bacteriocins-producing species among LAB include *L. rhamnosus*, *Lacticaseibacillus paracasei*, *Lactiplantibacillus pentosus* and *L. plantarum*, which synthesize antimicrobial compounds such as pentocin, helveticin, and plantaricin [82,83,84,85].

Within the *Bifidobacterium* genus, several bacteriocins have also been identified. For instance, *B. longum* subspecies are known to produce compounds such as bifidococcin, bisin, and bifidin I, while *B. bifidum* NCFB 1454 produces bifidin B [86,87]. However, these represent the only bacteriocins within this genus that have been partially or fully characterized to date. Other compounds reported in the literature are largely putative or have been identified based on genomic homology, including bifilong, bifilact Bb-12/Bb-46, and thermophilicin B67. Table 1 provides an overview of the postbiotics produced by LAB and Bifidobacteria, as reported in the recent literature.

### 2.3. Neurotransmitter Synthesis: γ-Aminobutyric Acid (GABA), Serotonin, Acetylcholine and Dopamine Production by Probiotic Bacteria

LAB and Bifidobacteria can synthesize or modulate several key neurotransmitters and neuroactive compounds, like GABA, Ach, serotonin, and dopamine. However, their effects on the CNS must be interpreted cautiously, as microbial neurotransmitters rarely cross the blood–brain barrier (BBB) in significant quantities. Instead, their influence is often indirect, occurring through local ENS signaling, enteroendocrine activation, immune modulation, or altered precursor availability.

Among these neuroactive molecules, GABA is the most extensively characterized. As the principal inhibitory neurotransmitter in the mammalian brain, GABA plays a crucial role in regulating anxiety, stress responses, relaxation, sleep, and emotional balance [92]. Several LAB and *Bifidobacterium* species, including *L. plantarum*, *L. delbrueckii* ssp. *bulgaricus*, *L. fermentum*, *L. brevis*, *B. adolescentis* and *Bifidobacterium dentium* possess glutamate decarboxylase systems that catalyze the conversion of glutamate into GABA [92,93,94]. In particular, a recent in vitro study explored the approach behind this capability and identified the strain *L. brevis* as a high-yield producer of GABA, providing a molecular basis that explains the efficiency of these bacteria in converting glutamate [95]. Furthermore, another study demonstrated this ability of *L. plantarum* through in vitro and in vivo experiments conducted with *Caenorhabditis elegans* nematode [96].

This process, mediated by a pyridoxal-5-phosphate-dependent enzyme and a glutamate/GABA antiporter, involves the decarboxylation of L-glutamate to GABA by the enzyme glutamate decarboxylase (GAD), typically expressed under acidic stress conditions [92,95,96,97]. In the host, GABA acts primarily through GABA-A and GABA-B receptors to reduce neuronal excitability [98].

Ach, a key neurotransmitter in both the central and peripheral nervous systems, plays a fundamental role in cognition, memory, and learning processes [99]. Certain LAB strains, such as *L. plantarum* AM2 and *L. reuteri*, have been shown to produce ACh under optimized conditions, as evidenced in an in vitro study that adopted a biotechnological optimization approach [99,100]. ACh is synthesized from choline and acetyl-CoA by choline acetyltransferase (ChAT) at presynaptic terminals and exerts a wide range of physiological effects, including vasodilation, stimulation of gastrointestinal peristalsis, modulation of bladder function, and contributions to muscle contraction, immune regulation, and cognitive function [101].

The relationship between LAB and Serotonin is largely indirect but biologically significant. Serotonin, which regulates mood, sleep, appetite, and gut motility, is primarily produced by enterochromaffin cells in the gastrointestinal tract. LAB can influence serotonin levels by modulating tryptophan metabolism, as well as through the production of Short-chain fatty acids and interactions with enteroendocrine signaling pathways [102,103,104].

For instance, in the piglet gut, *Lactobacillus amylovorus* has been shown to stimulate tryptophan hydroxylase 1 expression and enhance serotonin synthesis, at least partly through acetate signaling via free fatty acid receptor 3 [105].

Similarly, *L. reuteri* has been reported to upregulate enzymes involved in Serotonin biosynthesis while suppressing the kynurenine pathway in both the colon and prefrontal cortex. This shift may redirect tryptophan metabolism toward serotonin production rather than toward stress-associated metabolites. Notably, these effects appear to be strain-specific rather than a universal property of all LAB.

The influence of LAB on dopamine is even more indirect. While some gut bacteria possess enzymatic systems capable of converting catecholamine precursors such as levodopa into dopamine, this activity has been more extensively documented in *Enterococcus* species (*E. faecium* and *E. faecalis*) than in typical probiotic LAB strains [106].

Instead, LAB are thought to modulate dopaminergic signaling indirectly by altering precursor availability, reducing inflammation, and influencing stress-related hormonal pathways and gut microbial composition [107]. For example, clinical evidence demonstrated that *L. plantarum* DR7 reduces stress and anxiety in patients, modulating the expression of key enzymes involved in catecholamine synthesis, including dopamine β-hydroxylase and tyrosine hydroxylase [108]. Similarly, evidence from animal models suggests that treatment with *L. plantarum* PS128 [109] and certain probiotic formulations [110] has demonstrated beneficial effects on dopaminergic pathways and promotes neuronal protection. These effects are often associated with increased SFCAs levels, particularly butyrate, and reduced expression of monoamine oxidase-B, rather than direct microbial production of dopamine [109,110]. Overall, LAB do not necessarily act as direct sources of bioavailable dopamine; instead, they modulate the intestinal and systemic environment in ways that influence dopamine synthesis, turnover, receptor signaling, and overall catecholamine balance. In summary, the strongest mechanistic evidence supports LAB-associated GABA production, whereas effects on serotonin and dopamine are primarily mediated through host–microbe metabolic interactions and signaling pathways, rather than direct neurotransmitter delivery to the brain. Accordingly, claims regarding the neuroactive effects of these bacteria should be interpreted with appropriate caution and nuance.

## 3. Probiotic Bacteria in Metabolic Regulation

The human body harbors a vast and complex microbial community, comprising approximately 38 trillion bacterial cells [111]. The majority of these microorganisms reside in the gastrointestinal tract, where they form a highly dynamic ecosystem known as the gut microbiota. Although some bacterial species are pathogenic, most are beneficial. Collectively referred to as the Microbiome, these microorganisms play essential roles in digestion, immune regulation, and metabolic homeostasis [112]. The metabolic and regulatory effects of LAB and Bifidobacteria are multifaceted, arising from both enzymatic activities and direct physical interactions within the gastrointestinal tract. One key mechanism involves bile salt hydrolase (BSH) activity, which deconjugates bile acids and promotes their excretion. This process triggers a compensatory hepatic response, whereby the liver converts circulating cholesterol into new bile acids, thereby contributing to the reduction in serum cholesterol levels [113]. In addition, bacterial-derived enzymatic activities have been linked to blood pressure regulation and overall cardiovascular health. Emerging evidence suggests that BSH-producing bacteria can also reshape the composition of the gut microbiota. For example, increased BSH activity has been associated with a higher abundance of *B. pseudolongum*, which may further influence host metabolism through modulation of the Farnesoid X receptor signaling pathway. This interaction can enhance cholesterol catabolism and excretion [113]. Moreover, by reducing metabolic endotoxemia, these mechanisms help limit chronic low-grade inflammation, thereby decreasing the risk of atherosclerotic plaque formation and supporting cardiovascular health.

### 3.1. Modulation of Lipid Metabolism and Cholesterol Homeostasis

According to the World Health Organization (WHO), cardiovascular diseases (CVDs) account for nearly 18 million deaths annually, making them a leading category of non-communicable diseases (NCDs) [114]. Probiotics have emerged as promising agents for supporting cardiovascular health, primarily through modulation of the gut microbiota and the production of antibacterial compounds that help maintain intestinal homeostasis [115]. Several mechanisms underlie their lipid-lowering effects [113]. One key pathway involves the BSH enzyme produced by these microorganisms. BSH deconjugates bile salts, reducing their intestinal reabsorption. As a result, the liver increases the utilization of circulating cholesterol for de novo bile acid synthesis, thereby lowering serum cholesterol levels [116]. Another important mechanism is mediated by EPS. These polymers can bind cholesterol within the intestinal lumen, promoting its fecal excretion.

In addition, EPS and their derived monomers, such as glucose and guluronic acid produced by *L. fermentum* Y57, have been shown to inhibit both angiotensin-converting enzyme (ACE), a blood pressure regulator, and 3-hydroxy-3-methylglutaryl-CoA (HMG-CoA) reductase [113,117]. Inhibition of HMG-CoA reductase reduces endogenous cholesterol synthesis [118], contributing to improved lipid profiles. As demonstrated in an in vivo study, the effect of *L. fermentum* Y57 on the blood lipid profile also included a decrease in low-density lipoprotein (LDL) cholesterol and an increase in high-density lipoprotein (HDL) cholesterol levels [119]. Dysregulation of these pathways can lead to metabolic disorders, including hypercholesterolemia [118]. Emerging evidence further indicates that specific bacterial strains can upregulate peroxisome proliferator-activated receptors (PPARα and PPARγ), which play central roles in lipid metabolism. PPARα promotes fatty acid β-oxidation, whereas PPARγ regulates lipid storage and adipogenesis. Notably, the strain *L. amylovorus* CP1563 has been shown to strongly activate both receptors in obese mouse models [120], leading to reduced fatty acid synthesis, enhanced triglyceride breakdown, and increased β-oxidation and ameliorated dyslipidemia [120]. Interestingly, this strain exerts beneficial effects even in a non-viable (paraprobiotic) form, suggesting that inactivated bacteria may also hold therapeutic potential.

*B. longum* BB536 has been reported to reduce total cholesterol levels, liver lipid deposition, and adipocyte size [121] and to have an anti-obesity effect on high-fat-diet-induced obese rats [122]. The proposed mechanism is primarily linked to the production of acetic acid as a final metabolite, which improves glucose tolerance, increases insulin sensitivity, reduces obesity and metabolic abnormalities, and decreases the expression of genes involved in fat synthesis in the liver.

**Figure 2:**
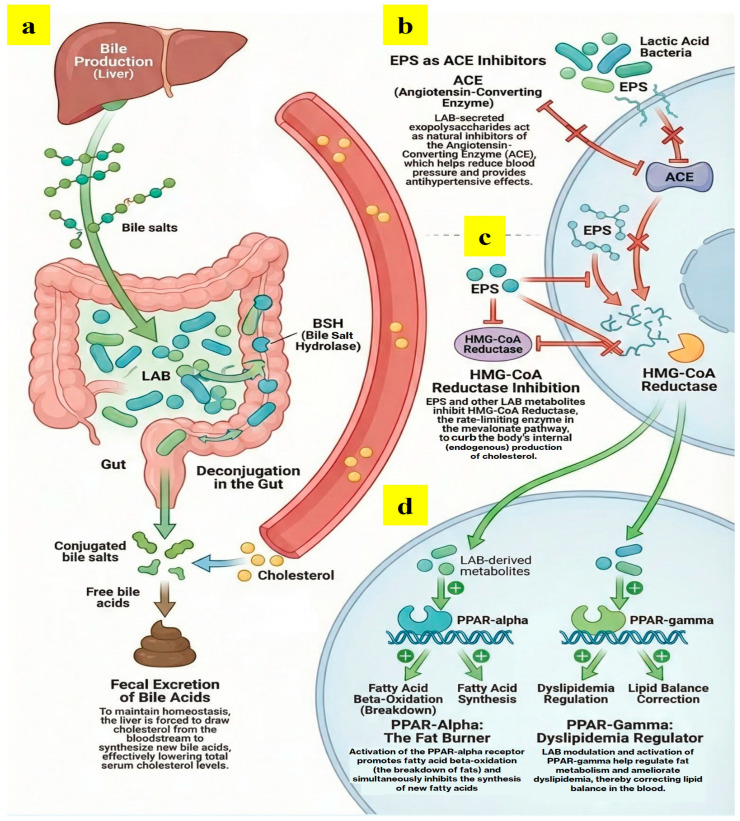
Overview of the lipid metabolism and cholesterol metabolic pathways of LAB (**a**) BSH Activity [116] (**b**) LAB-secreted EPS acting as an ACE inhibitor for antihypertensive effects [124] (**c**) Inhibition of HMG-CoA Reductase by EPS [113,118] (**d**) Activation of PPARα and modulation of PPAR-γ [120].

*Bifidobacterium lactis* IDCC 4301, instead, inhibits cell differentiation and lipid accumulation by suppressing the expression of adipogenic enzymes in 3T3-L1 cells, a standard model for studying the formation of body fat and then in high-fat diet-fed mice model [123]. Figure 2 illustrates the diverse mechanisms through which LAB regulate lipid metabolism and maintain cholesterol homeostasis.

### 3.2. Influence on Glycemic Control and Insulin Sensitivity

Diabetes is a chronic metabolic disorder and a major NCD, characterized by hyperglycemia, resulting from impaired insulin secretion, insulin action, or both. Insulin, produced by the pancreas, is essential for blood glucose regulation; its dysfunction leads to persistent hyperglycemia and progressive damage to blood vessels and nerves [125]. The glycemic index (GI) ranks carbohydrate-rich foods according to their effect on postprandial blood glucose levels (scale 0–100) [126]. Diets rich in high-GI foods are associated with rapid glucose spikes and increased risk of insulin resistance (IR). Furthermore, certain probiotic strains stimulate the secretion of glucagon-like peptide-1 (GLP-1) from intestinal L-cells [127]. GLP-1 improves glycemic control by suppressing glucagon release from pancreatic α-cells, slowing gastric emptying, and reducing the rate of intestinal glucose absorption. It also promotes satiety via CNS signaling, thereby supporting body weight management. For this reason, there is growing interest in developing low-GI foods, such as *Aloe Barbadensis*-based foods, to improve glycemic control and enhance insulin sensitivity as demonstrated in animal models [128]. The combination of LAB and Bifidobacteria in low-GI foods causes a modification in carbohydrate structures by converting raw material into simpler sugars, as demonstrated in an in vitro method [126], sugar alcohols, organic acids, short-chain fatty acids (SCFAs), and bioactive compounds such as GABA.

LAB, particularly species of Streptococcaceae and Lactobacillales, but also *Bifidobacterium* spp., can positively influence the glycemic properties of foods and host lipid and glycemic metabolism [129,130,131]. During fermentation, LAB modify carbohydrate structures by converting starch into simpler sugars, sugar alcohols, organic acids, SCFAs, and bioactive compounds such as GABA. These changes contribute to lower GI values and enhanced insulin sensitivity [125].

They also ferment undigested dietary fibers to produce SCFAs, which improve insulin sensitivity and glucose homeostasis [131] by activating free fatty acid receptors, also called G-protein-coupled receptors FFAR2 (GPR43) and FFAR3 (GPR41) [132]. In the liver, SCFA signaling via FFAR3 reduces hepatic glucose production through inhibition of gluconeogenesis [133]. In pancreatic β-cells, instead, FFAR2 activation enhances insulin secretion and promotes glucose uptake [132,134].

As regards *Bifidobacterium* species, in a randomized, double-blind, placebo-controlled trial, 8-week supplementation with *B. breve* BR03 and B632 led to a significant improvement in insulin sensitivity indices (QUICKI and ISI) in children and adolescents with obesity, by influencing the production of SCFAs, intestinal permeability and the pathways involved in glucose homeostasis and systemic inflammation [135]. The same applies to strains such as *B. breve* BR03 and *B. breve* B632 [136]. On the other hand, *B. adolescentis* exerts its metabolic effects by modulating the quantity and function of the gut microbiota, thereby reducing intestinal permeability and lipopolysaccharide (LPS)-induced endotoxemia, and attenuating the activation of pro-inflammatory pathways associated with insulin resistance [137].

Collectively, these mechanisms underscore the multifaceted role of LAB and Bifidobacteria in modulating carbohydrate metabolism and supporting metabolic health in diabetes.

### 3.3. Impact on Inflammation and Oxidative Stress

A large proportion of the body’s immune cells reside in the gastrointestinal tract, where they closely interact with the gut microbiota [138]. Dysbiosis can trigger the release of pro-inflammatory cytokines, contributing to neuroinflammation and increasing the risk of mood disorders and neurodegenerative diseases [139]. Probiotic species belonging to Lactobacillaceae and Bifidobacteriaceae exhibit strong immunomodulatory properties. They regulate immune responses by modulating the balance between pro-inflammatory cytokines (such as tumor necrosis factor-alpha (TNF-α), Interleukin-1 (IL-1), Interleukin-2 (IL-2), Interleukin-6 (IL-6), Interleukin-1β (IL-1β), Interleukin-12 (IL-12) and Interleukin-8 (IL-8)) and anti-inflammatory cytokines (such as Interleukin-10 (IL-10) and transforming growth factor beta (TGF-β)) [140,141]. These effects are mediated both by interactions with the gut microbiota and by direct probiotic–host cell contact. LAB interact with intestinal epithelial cells and dendritic cells (DCs) through pattern recognition receptors (PRRs) (e.g., Toll-like receptor 2 (TLR2), Toll-like receptor 4 (TLR4), and nucleotide-binding oligomerization domain containing 2 (NOD2)), influencing T-helper cell polarization (Th1, Th2, Th17), regulatory T cells (Treg) and downregulating nuclear factor kappa B (NF-Κb) and mitogen-activated protein kinase (MAPK) signaling pathways, thereby promoting immune cell recruitment and pathogen clearance [141,142,143].

Several specific strains have shown promising anti-inflammatory effects. *L. plantarum* A41, when combined with *L. fermentum* SRK414, significantly reduced pro-inflammatory cytokines, like TNF-α, IL-1β, and IL-8 while increasing IL-10 level [144,145]. Similarly, *L. plantarum* Lp91 attenuated colonic inflammation in murine models by decreasing TNF-α, Cyclooxygenase-2 (COX-2), and Interleukin-4 (IL-4) expression [146]. *L. rhamnosus* GG ATCC 7469 inhibited TNF-α production via TLR2-dependent mechanisms [147], whereas *L. paracasei* CNCM I-4034 promoted TGF-β secretion and reduced pro-inflammatory mediators in DCs [148]. In addition, specific structural components of LAB contribute to their immunomodulatory activity. For instance, the surface layer protein Slp-A from *L. helveticus* MIMLh5 and *L. acidophilus* inhibited NF-κB activation and IL-8 secretion through PRRs, while upregulating the anti-inflammatory regulator PPAR-γ [132,149]. Beyond inflammation, LAB also play a role in mitigating oxidative stress. They enhance the host antioxidant defense system by increasing the activity of enzymes such as superoxide dismutase (SOD), catalase (CAT), and glutathione peroxidase (GSH-Px), while reducing the accumulation of reactive oxygen species (ROS). This antioxidant capacity further contributes to the prevention of chronic inflammation and related metabolic and neurodegenerative disorders.

Certain LAB strains also produce bacteriocins (e.g., nisin, enterocin, plantaricin) that contribute to immunomodulation by suppressing pro-inflammatory signaling and enhancing IL-10 production [150,151,152].

In addition to immune modulation, probiotic LAB counteract oxidative stress through multiple mechanisms. They enhance the activity of endogenous antioxidant enzymes (SOD, CAT, GSH-Px), activate the Nuclear Factor Erythroid 2–Related Factor 2 (Nrf2) pathway, scavenge free radicals, and stimulate the production of SCFAs via microbiota modulation [153,154,155]. These actions reduce lipid peroxidation, limit reactive oxygen species (ROS) overproduction by inhibiting NADPH oxidase, and protect mitochondrial function [156,157]. Notable examples include *L. paracasei* NM-12 (high SOD and GSH-Px activity), *L. plantarum* GXL94, and *L. fermentum* JX306, which upregulate antioxidant genes and increase total antioxidative capacity under oxidative stress [131,156,158,159]. Furthermore, EPS on the LAB cell surface provide an additional protective effect by stimulating catalase production and reducing oxidative damage to cellular components [157,160,161].

As regards *Bifidobacterium* species, in a double-blind, randomized clinical trial involving patients with metabolic syndrome, *B. lactis* HN019 led to a significant reduction in levels of homocysteine, hydroperoxides and the pro-inflammatory cytokine IL-6, an increase in levels of adiponectin (a protein with anti-inflammatory and insulin-sensitizing activity) and nitric oxide (NOx) metabolites, suggesting an improvement in endothelial function. This happens through diverse mechanisms, for example by acting directly on adipocytes, increasing the expression of PPAR-alpha, inhibiting the production of TNF-alpha and the signaling of the nuclear factor NF-kB, and expressing the enzyme MnSOD (manganese superoxide dismutase) [162].

The experimental study conducted by Vitheejongjaroen et al. [163] evaluated the antioxidant and immunomodulatory properties of the probiotic strain *B. animalis* MSMC83 in a mouse model of accelerated ageing induced by D-galactose (D-gal), which triggers an overproduction of reactive oxygen species (ROS), resulting in lipid peroxidation, liver dysfunction and disruption of gut microbiota homeostasis. At the systemic and hepatic levels, a significant increase in the activity of endogenous antioxidant enzymes was observed, including SOD, CAT, and GSH-Px. Concurrently, a marked reduction in levels of malondialdehyde (MDA), a critical indicator of oxidative damage to cell membranes, was recorded. From an inflammatory perspective, there was a down-regulation of TNF-alpha, suggesting a synergistic action between the reduction in oxidative stress and the mitigation of the inflammatory response. The association between LAB and Bifidobacteria in reducing inflammation and oxidative stress was also assessed. In a study conducted on female BALB/c mice with ulcerative colitis, the administration of *L. plantarum* ZDY2013 and *B. bifidum* WBIN03 improved the integrity of the intestinal mucosa by modulating the immune response and reducing oxidative stress. In particular, the probiotics activated the Nrf2/ARE pathway, increasing the expression of antioxidant enzymes such as SOD and GSH-Px and reducing levels of MDA, a marker of lipid peroxidation. Concurrently, a down-regulation of the pro-inflammatory cytokines TNF-α and IL-6 and an increase in IL-10 were observed, associated with a partial restoration of the balance of the gut microbiota [164].

## 4. Gut-Microbiota-Brain Axis: The Psychobiotics Frontier

Specific microbial disturbances have been consistently linked to a wide range of psychiatric disorders, including depression and anxiety [165]. These findings provide compelling evidence that gut microbiota play a significant role in shaping the body’s stress-regulation system. The emergence of the psychobiotic concept has fundamentally transformed our understanding of MGBA. What was once viewed as a simple digestive relationship is now recognized as a complex, multi-channel regulatory network that influences mental health and neurological resilience. The traditional definition of “Psychobiotics” dates back to 2013, when Dinan et al. [166] described them as a subclass of probiotics with beneficial mental health effects, particularly in the context of psychopathology, when consumed in appropriate amounts [166]. Today, an expanded version of the definition is used, which includes any exogenous substances, such as prebiotics, postbiotics, and heat-killed paraprobiotics, which promote the growth of psychobiotic strains in the gut by interacting with the human gut microbiota [167,168].

This interaction operates through five primary communication pathways: neural, endocrine, immune, metabolic, and nutritional [23]. The neural pathway is primarily mediated by the vagus nerve, which serves as a direct electrophysiological link between the ENS and the brainstem [169,170,171]. This connection allows for rapid, bidirectional communication between the gut and the brain, influencing emotional and physiological states. The endocrine pathway exerts its effects through the regulation of the hypothalamic–pituitary–adrenal (HPA) axis, which, under chronic psychosocial stress, becomes hyperactive [172]. Psychobiotics have been shown to modulate this response, acting as a biological “brake” that restores hormonal balance [173]. This hormonal regulation also influences sleep architecture. Clinical trials have demonstrated that psychobiotics can increase rapid eye movement (REM) sleep duration and reduce wake after sleep onset (WASO), suggesting that gut-derived signals play a role in recalibrating the brain’s circadian rhythm and stress-recovery mechanisms.

On the immune front, psychobiotics strengthen intestinal tight junction integrity and promote the production of anti-inflammatory cytokines [138,139]. By shifting the systemic environment from pro-inflammatory to an anti-inflammatory state, they help protect the brain from cytokine-driven neuroinflammation, which is often linked to psychological distress and cognitive decline.

The metabolic and nutritional pathways further highlight the precision of the MGBA [174]. Through fermentation of prebiotic fibers, gut bacteria produce SCFAs, which function as potent epigenetic modulators [70,71]. These molecules influence histone acetylation and the expression of brain-derived neurotrophic factor (BDNF), a protein critical for neuronal plasticity and survival.

Recent research has extended these insights to neurodegenerative diseases (NDDs), such as Alzheimer’s, Parkinson’s, and Amyotrophic Lateral Sclerosis (ALS) [171,175]. Microbial metabolites classified as postbiotics, such as indole-3-propionic acid (IPA) and the tauroursodeoxycholic acid (TUDCA), have been shown to cross the blood–brain barrier and exert antioxidant and neuroprotective effects. These metabolites promote mitophagy and help prevent protein misfolding in aging neurons, offering potential therapeutic benefits. Direct postbiotic supplementation represents a more targeted therapeutic approach, particularly for immunocompromised patients, where live microbes may exhibit unpredictable effects. This strategy provides a more controlled and precise method of modulation, bypassing the potential variability of live probiotic interventions.

Ultimately, modulating bacteria-gut–brain signaling represents a paradigm shift in neuroscience. By targeting the gut as a primary site for psychological and neurological intervention, it is possible to develop integrated treatments that address the systemic roots of mental illness and foster neurological resilience originating from the microbiome.

Table 2 provides a summary of these mechanisms, elucidating the complex biological processes that link microbial ecology with neurological function.

### 4.1. Intestinal Barrier Integrity (The “Leaky Gut” Connection to Systemic Health)

**Figure 3:**
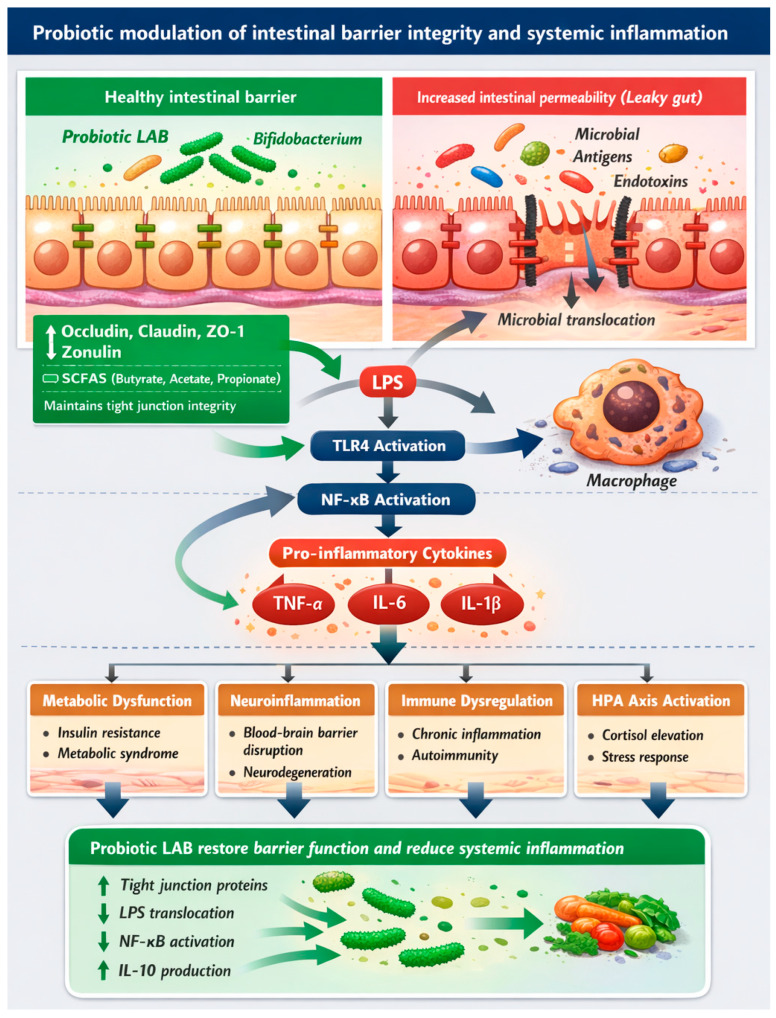
Mechanistic links between intestinal barrier dysfunction, endotoxin translocation, and downstream systemic effects [184]. Abbreviations: ↑, increased; ↓, decreased.

The intestinal epithelial barrier is fundamental in preventing the translocation of microbial products, such as lipopolysaccharides (LPS) and pro-inflammatory antigens, into the systemic circulation [15]. Increased intestinal permeability, commonly known as leaky gut syndrome (LGS), also allows luminal antigens, bacteria, and endotoxins to cross the barrier, triggering metabolic endotoxemia and chronic low-grade systemic inflammation [177]. This gut-derived leakage activates TLR-4 and NF-κB signaling pathways, leading to elevated pro-inflammatory cytokines (TNF-α, IL-6, IL-1β), HPA axis hyperactivity, insulin resistance (IR), endothelial dysfunction, neuroinflammation, and immune dysregulation. These processes contribute to the pathogenesis of numerous NCDs, including metabolic syndrome, type 2 diabetes, obesity, non-alcoholic fatty liver disease, and MDD, such as depression [178,179,180,181,182,183,184,185,186,187]. Probiotic species belonging to the Lactobacillaceae and Bifidobacteriaceae families reinforce the intestinal barrier by upregulating tight junction proteins such as occludin, claudins (e.g., claudin-1, -3), and zonula occludens-1 (ZO-1), and downregulating zonulin, a protein that increases paracellular permeability [188,189,190] (Figure 3).

These effects are mediated by direct bacterial components, including SLPs and EPS, which are naturally produced during the fermentation of milk, vegetables, or grains [191,192,193].

Multi-strain probiotic formulations have shown promising results in restoring intestinal barrier integrity [131]. However, due to high variability in study outcomes, further research is needed. Future randomized controlled trials should employ standardized methodologies, clearly defined strains and dosages, and longer intervention periods to better understand the underlying mechanisms and support personalized clinical applications [194,195,196].

### 4.2. The Enteric Nervous System and Vagus Nerve Signaling

The ENS, often referred to as the “second brain”, and the vagus nerve serve as primary pathways through which probiotics can influence brain function. The ENS, located within the gut wall, regulates several key processes, including intestinal motility, secretion, blood flow, barrier function, and local reflexes. It maintains a dense, bidirectional communication with the vagus nerve—the main parasympathetic connection between the gut and brain regions involved in emotional and stress regulation [197,198,199]. This intricate network allows for direct influence of gut microbiota on brain function, highlighting the critical role of the gut–brain axis in modulating mental health and physiological responses.

Vagal afferent fibers transmit sensory information from the gut to the brainstem. Microbial metabolites and neurotransmitters produced by LAB can activate enteroendocrine cells, enteric neurons, and vagal afferents, thereby modulating emotional and cognitive responses [107,169,170,200].

**Figure 4:**
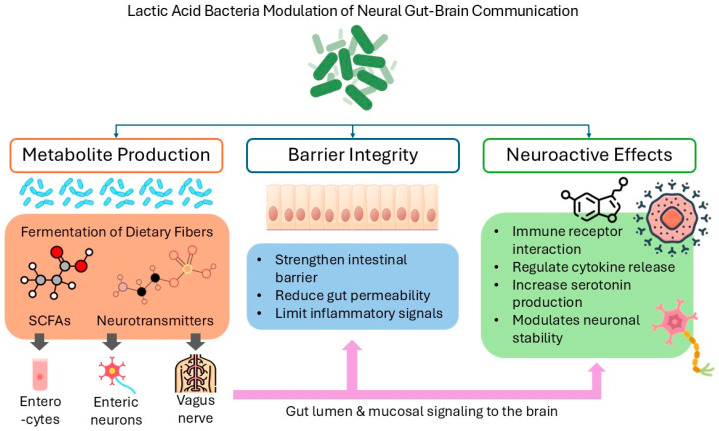
A schematic illustration of the mechanisms through which LAB influence gut–brain communication by producing metabolites [107,200], strengthening the integrity of the intestinal barrier [201,202], and exerting neuroactive regulation [203,204].

Probiotics engage this gut–brain neural pathway through several mechanisms (Figure 4). First, fermentation of dietary substrates generates metabolites that stimulate enteroendocrine cells and vagal afferents [107,200]. Second, LAB strengthen the intestinal epithelial barrier, reducing permeability and limiting the passage of inflammatory signals that could impair ENS and vagal function [201,202]. Third, certain strains interact with pattern-recognition receptors on epithelial and immune cells, indirectly influencing neuroactive signaling via cytokine modulation, serotonin release, and changes in neuronal excitability [203,204]. LAB-derived metabolites, immune signaling, and vagal interactions collectively contribute to modulation of the gut–brain axis.

A key study demonstrated the importance of this pathway: *L. rhamnosus* JB-1 altered emotional behavior and brain GABA receptor expression in mice, but these effects were completely abolished by vagotomy, confirming that intact vagal signaling is required for the probiotic’s central effects [205,206].

Although this work was conducted in animals, it remains one of the most influential mechanistic demonstrations that a probiotic strain can signal to the brain through a neural route.

The ENS–vagus framework should not be interpreted as a simple one-strain, one-outcome model. These psychobiotic effects are highly strain-specific, depending on the strain’s ability to produce neuroactive metabolites, interact with the epithelium, and modulate gut–brain signaling [207]. Outcomes also vary according to host physiology, diet, baseline microbiota, and the delivery matrix of the probiotics [208,209]. While the ENS–vagus axis provides a direct neural bridge, other mechanisms, including immune modulation, microbial metabolites, and endocrine signaling, also contribute to the effects of LAB on mental health [210,211]. These complex and multifaceted interactions contribute to the overall therapeutic potential of probiotics in influencing mood, cognition, and neurological health.

### 4.3. Attenuation of HPA Axis Reactivity and Cortisol Regulation

Another pathway through which probiotics influence health is by modulating the HPA axis, the body’s central stress-response system. Upon stress exposure, the hypothalamus releases corticotropin-releasing hormone (CRH), which stimulates ACTH secretion from the pituitary gland. ACTH then prompts the adrenal glands to produce glucocorticoids, primarily cortisol [212]. Chronic HPA axis hyperactivity leads to sustained elevated cortisol levels, which are associated with sleep disturbances, cognitive impairment, and increased risk of anxiety and MDD.

Dysbiosis, increased intestinal permeability, and altered mucosal immunity can all contribute to HPA axis dysregulation via the MGBA [172]. Conversely, changes in gut microbiota composition can influence cortisol production and stress reactivity [176,213]. For this reason, LAB have gained attention as potential dietary interventions to buffer stress responses through gut–brain interactions [210,214].

The mechanisms underlying LAB-mediated HPA axis modulation are multifactorial. One well-supported route involves reinforcement of the intestinal epithelial barrier. Preclinical studies show that stress increases gut permeability and endotoxin translocation, whereas certain LAB strains, such as *Lactobacillus farciminis*, can attenuate these effects in animal models [215,216]. Another important mechanism is immune-mediated: microbial products such as LPS trigger pro-inflammatory cytokines (IL-1, IL-6, TNF-α) that activate the HPA axis. By reducing dysbiosis and inflammation, LAB can indirectly dampen this signaling [213].

Effects on tryptophan metabolism and short-chain fatty acid (SCFA)-mediated hypothalamic modulation have been proposed but remain less directly demonstrated in LAB-specific studies [210,217]. In humans, evidence is still limited and heterogeneous. Most clinical trials use multi-strain formulations (often combining LAB with Bifidobacteria), making it difficult to attribute effects to specific LAB strains [218]. Psychobiotic efficacy is highly strain-dependent and influenced by delivery format (capsules, fermented dairy, or food matrices), treatment duration, baseline stress levels, and host phenotype [219,220]. Overall, current data suggest that probiotic bacteria can contribute to attenuation of HPA axis hyperreactivity and improve stress resilience, but they should be considered adjunctive modulators rather than primary regulators. Future research should focus on strain-specific effects, standardized cortisol measurements, and well-characterized study populations to better define their role in mental health.

## 5. Clinical Evidence of Probiotic-Containing Foods in Health

The growing imbalance of the microbiota has been influenced by the widespread use of antibiotics, the emergence of antibiotic-resistant bacteria, and significant dietary changes associated with modern lifestyles. The viability and metabolic activity of these microorganisms within the gastrointestinal tract are essential for ensuring their beneficial health effects [221]. However, one of the major challenges in the application of probiotics in the food industry is their sensitivity to heat treatments during food processing, as well as to the harsh conditions of the human gastrointestinal tract [222]. Probiotic survival is critical, as these microorganisms must withstand gastric acidity and bile salts, remain viable both in the final food product and after ingestion, and adhere to the intestinal mucosa to exert their beneficial effects. To overcome these limitations, researchers and the food industry are exploring novel technologies and innovative delivery strategies [223]. These challenges have encouraged food manufacturers to develop innovative probiotic-enriched products. The effectiveness of probiotics depends on several factors, including the specific strain used, the administered dose, the duration of supplementation, the health status of the consumer, and the delivery system, such as dairy products, tablets, or capsules. In particular, the food matrix plays a crucial role in maintaining probiotic viability, functionality, and stability in functional foods, while also influencing sensory quality, consumer acceptance, and the overall efficacy of the final product. Although probiotics are generally regarded as beneficial, they may occasionally cause mild and temporary side effects, primarily gastrointestinal symptoms such as bloating, diarrhea, constipation, and nausea, especially in immunocompromised individuals or transplant recipients [221,224]. In these cases, the potential benefits should be carefully weighed against the possible risks [225,226].

Overall, these findings highlight the importance of conducting long-term, randomized clinical trials using standardized protocols to identify the most effective probiotic strains, dosages, and administration methods, thereby strengthening the scientific evidence supporting the health benefits associated with probiotics.

### 5.1. Probiotics in Foods

Fermented foods represent a practical and effective vehicle for delivering probiotics and their bioactive metabolites. These products include traditional dairy-based foods such as fermented milk, ice cream, yogurt, frozen yogurt, kefir, and cheese, as well as an expanding range of plant-based alternatives derived from cereals, pseudocereals, legumes, fruits, vegetables, and tree nuts.

The probiotic strains most frequently used in these products include *L. plantarum*, *L. rhamnosus* GG, *L. delbrueckii* ssp. *bulgaricus*, *S. thermophilus*, *B. breve*, *B. longum*, *B. lactis*, and *L. casei*. These microorganisms may be incorporated alone or in combination with starter cultures, contributing not only to probiotic functionality but also to improvements in flavor, texture, and nutritional quality [227,228,229,230,231,232,233,234,235,236,237,238,239,240,241,242,243,244,245,246,247,248,249,250,251,252,253,254,255,256,257,258,259,260,261,262,263,264,265,266,267,268,269,270,271,272,273,274,275,276,277]. A detailed overview of the principal food matrices used for probiotic foods and beverages, together with information regarding probiotic viability under different storage conditions, is presented in Table 3.

Dairy products remain the most established probiotic carriers because their buffered pH, protein and fat content, and nutrient composition promote microbial survival during processing, storage, and gastrointestinal transit. Fermented dairy products such as yogurt, kefir, fermented milk, cheese, and probiotic ice cream continue to dominate the functional food market because of their high consumer acceptance and demonstrated efficacy [221,269].

Cheese, particularly fresh or soft varieties such as “Ricotta” and “Scamorza”, offers especially favorable conditions for probiotic stability because of its low oxygen content, relatively stable pH, and lipid-rich matrix. Strains including *B. animalis* subsp. *lactis*, *L. acidophilus*, *L. casei*, and *L. rhamnosus* have maintained high viability levels up to 10^9^ CFU/g during refrigerated storage and ripening [233,234,235,236,237]. Similarly, frozen dairy products can preserve probiotic viability, particularly when supplemented with cryoprotectants or prebiotic compounds such as inulin, lactitol, and fructo-oligosaccharides (FOS). Yogurts enriched with inulin or green banana pulp, for example, have maintained probiotic counts between 10^6^ and 10^8^ CFU/g after 30–45 days of storage [232], while cheeses such as cheddar and cottage cheese have shown viable counts of 10^8^–10^9^ CFU/g [234,235].

Interest in non-dairy probiotic foods has grown rapidly in response to increasing demand for vegan, lactose-free, and sustainable products. Plant-based matrices derived from oats, rice, quinoa, soy, almonds, cashews, legumes, fruits, and vegetables have demonstrated the ability to support probiotic growth and survival. Cereals and pseudocereals are particularly attractive because they naturally contain prebiotic fibers, β-glucans, and bioactive compounds that enhance probiotic functionality [278,279]. For example, co-fermentation of oat flour with *L. plantarum* improved probiotic viability, highlighting the potential of cereal-based functional beverages. However, plant-based matrices generally exhibit lower buffering capacity and different protein and lipid compositions than cow’s milk, making probiotic stability more challenging and requiring targeted technological strategies. Among these strategies, microencapsulation has emerged as one of the most effective approaches for protecting probiotics during food processing, storage, and gastrointestinal transit. Encapsulation systems based on alginate, chitosan, and resistant starches improve bacterial resistance to environmental stress [280]. For instance, microencapsulated *L. acidophilus* incorporated into bread formulations retained satisfactory viability after storage, while fermented oat- or rice-based beverages supplemented with inulin and encapsulated probiotics maintained viable counts above 10^7^–10^8^ CFU/mL for up to 49 days at 4 °C.

Fruit and vegetable juices also represent promising probiotic carriers because of their high nutritional value and sensory acceptability. Juices from pomegranate, apple, pineapple, orange, and cornelian cherry are particularly rich in antioxidants and bioactive compounds. Despite the technological challenges posed by low pH and dissolved oxygen, selected strains such as *L. plantarum*, *L. casei*, and *P. acidilactici* have maintained viability levels above 10^7^–10^9^ CFU/mL during refrigerated storage, especially when combined with prebiotic fibers or microencapsulation technologies [262,263,264,265,266,267,268,269].

Another innovative strategy involves the incorporation of probiotics into edible coatings and bioactive films, which act as carriers that release viable microorganisms during consumption [269]. Biopolymers such as cellulose derivatives, alginates, pectins, chitosan, seaweed extracts, and zein have been widely investigated for this purpose. Films containing *L. acidophilus*, *L. casei*, *L. rhamnosus*, *B. bifidum*, or *L. plantarum* demonstrated good stability during refrigerated storage, maintaining viability for periods ranging from 7 to 60 days [281,282,283]. These edible probiotic systems have been successfully applied to products such as bread, strawberries, and blueberries, where they improved both shelf life and microbiological quality.

Overall, technologies such as microencapsulation, edible coatings, and bioactive films have proven effective in maintaining probiotic concentrations above 10^6^–10^7^ CFU/g or mL throughout product shelf life, while helping achieve the recommended daily intake of at least 10^9^ CFU/day [3]. The health benefits associated with probiotics are primarily linked to modulation of the gut microbiota, reinforcement of the intestinal barrier, and interactions with the immune system. Their best-documented effects concern gastrointestinal health, including prevention of antibiotic-associated, viral, and radiation-induced diarrhea, improvement of irritable bowel syndrome symptoms, support in inflammatory bowel disease management, and enhancement of lactose digestion.

Beyond gastrointestinal effects, probiotics also exhibit immunomodulatory, anti-allergic, and metabolic benefits. They can improve lipid profiles, reduce oxidative stress, and contribute to cholesterol reduction. For example, kefir consumption has been associated with reductions in total and LDL cholesterol, while LAB-rich fermented foods have increased HDL cholesterol levels [284,285,286]. Clinical studies in patients with chronic kidney disease have also reported improvements in triglyceride and cholesterol levels following supplementation with *Lactobacillus* and *Bifidobacterium* strains [131].

Emerging evidence further suggests that probiotics may contribute to cancer prevention, particularly colorectal cancer, through anti-inflammatory and pro-apoptotic mechanisms. In addition, probiotics participate in the synthesis of vitamins B and K and may influence the gut–brain axis. Growing interest has therefore focused on their potential role in mental health and psychobiotics. Although evidence remains preliminary, fermented probiotic foods, especially dairy and soybean-based products, have shown potential benefits for stress resilience, sleep quality, and cognitive performance. However, these effects appear highly strain-specific and dependent on dosage, food matrix, and population studied, highlighting the need for further clinical validation.

Collectively, these findings indicate that LAB and *Bifidobacterium* species are not merely passive components of the gut microbiota but metabolically active microorganisms capable of modulating pathways involved in gastrointestinal, immune, metabolic, and neuropsychological health. This supports their emerging role within the broader concept of psychobiotics and functional nutrition.

### 5.2. Effects on Mood and Cognitive Health

Specific LAB strains have shown promising effects on brain function and mental health when delivered through food or supplements. For example, when *L. rhamnosus* HN001 was combined with a milk fat globule membrane-enriched ingredient in an animal model, it reduced the expression of several GABA-A receptor subunits in amygdala and hippocampus—brain regions involved in fear, anxiety, and memory. However, no corresponding behavioral changes were observed, suggesting neurobiological modulation without clear functional benefits [287]. Among food-based interventions, fermented milk containing *L. casei* Shirota provides some of the most consistent clinical evidence (Table 4).

Daily consumption preserved gut microbiota diversity, alleviated stress-associated abdominal dysfunction, and improved sleep quality under psychological stress [288,289,290]. The same strain also enhanced Th1 immunity, phagocytic activity, natural killer (NK) cell function, and mucosal Immunoglobulin A (IgA) production in elderly subjects [44,287]. Other notable findings include the following. *L. helveticus* MIKI-020, administered in tablets to adult patients, improved sleep efficiency, subjective sleep quality, motivation, and calmness after 4 weeks in healthy adults [294].

*L. plantarum* DR7 and *L. paracasei* K56 reduced anxiety and stress symptoms, lowered plasma cortisol, decreased pro-inflammatory cytokines, increased anti-inflammatory cytokines, and modulated serotonin and dopamine levels. These changes were accompanied by improvements in attention, emotional cognition, and associative learning, particularly in adults over 30 years old [12,109,307,308].

*L. plantarum* C29, delivered via fermented soybean capsules, improved cognitive performance and attention in individuals with mild cognitive impairment, while increasing serum BDNF levels [293]. The gut and brain follow a circadian rhythm, which, when disrupted, leads to microbial imbalance, destabilization of the HPA axis, and increased neuroinflammation [309].

The multi-strain combination of *L. helveticus* R0052 and *B. longum* R0175 and *L. rhamnosus* JB-1 normalized HPA axis activity and reduced psychological distress in both preclinical models and clinical cohorts [296,310].

Yogurt fermented with *L. delbrueckii* ssp. *bulgaricus* OLL1073R-1 improved sleep quality and psychological quality of life (general health and vitality) in a large number of randomized trials [311], in addition to the already known effects on immunity [312].

The cognitive evidence appears somewhat stronger, particularly for fermented soybean-based interventions. *L. fermentum* A2.8, delivered through tempeh, improved memory, verbal fluency, and visuospatial performance in older adults with mild cognitive impairment, with greater effects at higher doses [292].

These results were obtained primarily through questionnaires (State-Trait Anxiety Inventory (STAI), Pittsburgh Sleep Quality Index (PSQI), Short Form Health Survey (SF-8), Gastrointestinal Symptom Rating Scale (GSRS), Oguri–Shirakawa–Azumi sleep, Visual Analogue Scale) to evaluate somatic symptoms, computerized tests (neurocognitive function tests (CNT) to measure cognitive impairment, screening tests specifically designed for older adults to assess depressive symptoms while excluding somatic ones, and self-report scales (SAS-SDS) that measure anxiety levels based on affective and somatic symptoms. Several clinical and preclinical studies have investigated the effect of Bifidobacteria on mental health, supporting the hypothesis of the gut–brain axis’s involvement. Akkasheh et al. [297] and Lee et al. [298] conducted clinical trials on healthy patients with MDD, administering a multi-species probiotic (e.g., *L. acidophilus*, *L. casei*, *B. adolescentis*, *L. reuteri*, *B. bifidum*), resulting in a significant reduction in depressive symptoms measured using the BDI scale and an improvement in sleep quality.

Pinto-Sanchez et al. [300] and Tian et al. [306], in clinical trials involving patients with Irritable Bowel Syndrome (IBS) and mild-to-moderate anxiety and depressive symptoms, demonstrated that *B. longum* NCC3001 significantly reduced depression and normalized hyperactivity in the amygdala and limbic regions. This was also observed by Okubo et al. [299] in an open-label study of patients with schizophrenia and anxiety-depressive symptoms, in which *B. breve* A-1 led to a significant improvement in anxiety and depression. By supplementing female taekwondo athletes with *B. animalis* ssp. *lactis* BB-12, a clear link was identified between a healthier gut microbiome and reduced mental exhaustion. After 8 weeks of treatment, athletic performance significantly improved thanks to the alleviation of mental fatigue, accompanied by upregulation of beneficial gut microbiota, inhibiting harmful microbes, and regulation of relevant metabolic pathways [302].

Another interesting pilot study randomized FGDI (functional gastrointestinal disorders) patients, who received for 4 weeks fermented milk containing *B. bifidum* YIT 10347. Psychological symptoms were evaluated using the Profile of Mood States (POMS) and Total Mood Disturbance (TMD) tools. At the end of the treatment, they showed the improvement of the gastrointestinal environment and psychological symptoms [303].

In 224 healthy adults, after probiotic yogurt supplementation (*L. gasseri* SBT2055 and *B. longum* SBT2928), the natural killer cells’ activities increased, while plasma and salivary levels of ACTH and cortisol reduced, providing health benefits not only by enhancing innate immunity but also by alleviating stress [304].

Further reinforcing the idea that probiotics have positive effects on mental health and the hypothalamic–pituitary–adrenal axis, probiotic yogurt and multispecies probiotic capsules supplementation improved mental health and emotional status. These effects were evaluated using the Mini-Mental State Examination (MMSE), the Psychological Experiments Construction Language Test Battery, the Beck Depression Inventory (BDI) and the State-Trait Anxiety Inventory (STAI) [305], after 10 weeks of treatment in healthy adults.

Collectively, these studies indicate that specific probiotic strains, whether delivered in dairy, soybean-based foods, or supplements, can positively influence stress resilience, sleep, immunity, and cognitive function through integrated neural, immune, and neurochemical pathways. However, most evidence comes from preventive settings in healthy or mildly impaired populations rather than clinical psychiatric cohorts. Moreover, many trials lack rigorous blinding or use multi-component matrices, making it difficult to isolate the specific contribution of the LAB strain from that of the food vehicle. Larger, well-controlled studies are needed to confirm efficacy and support broader clinical translation.

### 5.3. Immunomodulation: Balancing Pro-Inflammatory and Anti-Inflammatory Cytokines

Daily consumption of yogurt fermented with *L. delbrueckii* ssp. *bulgaricus* OLL1073R-1 significantly enhanced NK cell activity (up to 150%) and promoted Th1-biased immunity through elevated Interferon Gamma (IFN-γ) in elderly individuals [313]. These effects, largely attributed to the EPS, activate NK cells and DCs via TLR pathways, reducing incidence of common colds and upper respiratory tract infections [314,315]. *L. fermentum* CECT5716 modulates cytokine profiles, enhances immunoglobulin production, downregulates pro-inflammatory mediators in macrophages, and promotes regulatory T cell differentiation [316,317]. In preclinical colitis models, it attenuated histological damage, myeloperoxidase activity, and Th17 infiltration while increasing SCFA production and antioxidant levels [317,318]. Similarly, *B. breve* Bif11 downregulates pro-inflammatory mediators such as nitric oxide in lipopolysaccharide-stimulated macrophages and ameliorates dextran sodium sulfate-induced colitis by reducing colon shortening, spleen hypertrophy, histological damage, and systemic cytokine levels. It also elevates antioxidant enzymes (catalase and glutathione) and decreases lipid peroxidation [319].

Collectively, these studies demonstrate the strain-specific immunomodulatory potential of probiotic LAB, ranging from enhanced innate immunity to anti-inflammatory and antioxidant effects. Overall, current clinical evidence indicates that LAB-containing fermented foods are most effective as adjunctive strategies to support stress resilience, sleep quality, cognitive function, and chronic inflammatory disorders, rather than as stand-alone interventions for clinically diagnosed anxiety or depression.

Future research should prioritize larger, well-controlled trials with diverse populations, standardized clinical endpoints, clear strain attribution, and a broader exploration of non-dairy fermented matrices (such as kefir, kombucha, miso, and natto) to improve consumer acceptance and facilitate dietary integration of psychobiotics.

## 6. Challenges and Future Perspectives

The effectiveness of probiotics is limited by several factors, including poor survival in the gastrointestinal tract, substantial individual variability, and regulatory challenges. Most of the LAB consumed is destroyed by stomach acid and bile, meaning that only a small fraction reaches the colon alive. This reduction in viable bacteria diminishes the production of essential substances, such as short-chain fatty acids (SCFAs) and neuromodulators [320,321].

Probiotics’ effects are significantly host-specific, as the baseline microbiome composition, nutrition, and genetics influence whether a particular strain can successfully engraft and produce beneficial metabolites [322,323]. Recent advancements, such as metabolic modelling and multi-omics prediction pipelines, are aiding in the rational selection and customization of LAB therapy to improve its efficacy. While safety is generally adequate for conventional probiotic strains in healthy individuals, comprehensive guidelines for novel strains and their use in at-risk patients remain inconsistent [324]. This gap in regulatory frameworks underscores the need for more standardized safety assessments and personalized approaches to probiotic therapy [325].

The development of robust delivery systems, such as microencapsulation, and the use of stress-tolerant probiotic strains are recommended to enhance gastrointestinal survival [326]. In addition, implementing “precision probiotic” strategies that integrate microbiome profiling and AI models to match specific strains with individual hosts is crucial for optimizing therapeutic outcomes [327].

To further improve efficacy and safety, it is essential to standardize regulatory frameworks to include mandatory strain genomics, quality controls, and adverse event monitoring. These measures are supported by organizations such as the ISAPP and the United States Pharmacopeia (USP) [328].

Moreover, achieving therapeutic advantages in fields such as metabolism and psychiatry requires cohesive, multidisciplinary research efforts and evidence-based regulation. This approach will ensure that probiotics can be effectively and safely integrated into clinical settings, addressing the complexities of individual responses and maximizing their potential benefits [329].

### 6.1. Bioavailability and Survival in the Gastrointestinal Tract

A major challenge is the low survival rate of LAB during gastrointestinal transit. Most strains are inactivated by the low pH of the stomach (~1–3) and by bile salts and pancreatic enzymes in the small intestine, resulting in only a small fraction reaching the colon alive [319,320,330,331,332]. This significantly limits the production of beneficial metabolites such as SCFAs, GABA, and serotonin precursors.

Essential LAB survival strategies are strain-dependent and frequently inadequate [333,334], and are affected by intrinsic factors including acid tolerance, BSH activity, adhesion proteins, and exopolysaccharide synthesis [335,336]. Specific strains contain genes that provide resilience to acidic and bile conditions, allowing them to sustain metabolic activity and engage with host intestinal epithelial cells [337,338]. To overcome these barriers, advanced delivery technologies are being developed.

Probiotic microencapsulation is a crucial method for shielding probiotics from environmental stressors, thereby enhancing their stability and ensuring effective delivery to the gastrointestinal tract. This technique protects probiotic cells from the detrimental effects of gastric acidity and enzymatic degradation, thus improving their transport efficiency to the intestinal environment [339,340]. In addition, microencapsulation and co-formulation with prebiotics are also among established methods to regulate pH and improve the bioavailability of LAB [221] and incorporating novel approaches such as multilayer nano coatings and dual-core systems [341]. Microencapsulation using spray-drying or freeze-drying, multilayer coatings, and co-formulation with prebiotics have shown promise in protecting LAB from gastric acid and enzymatic degradation, thereby improving viability, targeted release, and bioavailability [339,340,341,342]. Additionally, biofilm formation and cell aggregation can further enhance colonization and stress resistance [343]. Obviously, future research is required to focus on the integration of systems biology approaches, advanced delivery technologies, in vivo validation models, and the incorporation of prebiotics.

### 6.2. Personalized Probiotics: Host-Specific Microbial Responses

The increasing recognition of inter-individual heterogeneity in gut microbiota composition has transitioned probiotic research from a traditional “one-size-fits-all” methodology to precision or personalized probiotics [344]. The efficacy of LAB is strongly host-specific, depending on baseline gut microbiota composition, genetics, diet, lifestyle, and immune status [345]. This heterogeneity explains why the same strain may produce robust effects in one individual but minimal benefits in another.

Progress in metagenomics, metabolomics, and multi-omics technologies, combined with machine learning, microbiome sequencing and predictive modelling, are paving the way for precision probiotics tailored to individual microbiome profiles [220,344,346,347,348,349,350,351], customizing probiotic formulations to specific metabolic or neurological conditions. Addressing these challenges requires comprehensive longitudinal studies and strong interdisciplinary collaboration to develop evidence-based, personalized probiotic interventions that can be reliably translated into clinical practice.

### 6.3. Regulatory Frameworks and Safety Profiles

As probiotic LAB transition from functional food to live biotherapeutic products, harmonized regulatory frameworks become essential [352,353,354]. Global regulatory approaches to probiotics exhibit considerable variation; for instance, the European Food Safety Authority (EFSA), the FDA, and numerous national regulatory agencies formulate distinct guidelines concerning the safety, effectiveness, and labelling of probiotic products [354].

The safety evaluation of probiotic LAB frequently involves an examination of hemolytic activity, antibiotic resistance patterns, pathogenic potential, and metabolic by-products [355]. Most clinically relevant LAB strains are classified as GRAS or QPS organisms, reflecting their long-standing record of safe use in fermented foods and dietary supplements [356,357]. However, concerns persist regarding antibiotic resistance gene transfer, opportunistic infections in immunocompromised individuals, and inconsistencies in commercial product quality [358].

Future progress will require standardized protocols for efficacy evaluation, rigorous clinical trials, and global alignment of regulatory standards, including mandatory genomic safety data and adverse-event monitoring, as recommended by ISAPP and USP [328]. A further regulatory challenge stems from the lack of standardized protocols for evaluating probiotic effectiveness, particularly concerning metabolic and mental health outcomes. Diverse clinical studies employ different strains, dosages, and experimental designs, thereby hindering the development of consistent standards for therapeutic use [359]. Consequently, future regulatory frameworks may necessitate the incorporation of detailed microbiome data, strain-specific functionalities, and robust clinical evidence to support the progression of next-generation probiotic therapies. Ultimately, the alignment of global regulatory policies and the execution of rigorous clinical trials, genomic safety assessments, and standardized manufacturing processes will be essential for the secure and effective transformation of probiotic LAB into therapeutic interventions for human health.

## 7. Conclusions

This review highlights the significant contribution of LAB to metabolic health, particularly their ability to regulate cholesterol levels, improve glycemic control, and attenuate inflammatory processes. LAB are more than beneficial microorganisms present in fermented foods; they play a crucial role in linking dietary intake to systemic metabolic and neurological function. These effects are largely mediated by bioactive metabolites, especially short-chain fatty acids (SCFAs), which help maintain intestinal homeostasis and influence key metabolic pathways throughout the body.

Beyond metabolic regulation, LAB are increasingly recognized for their potential role in neurophysiological processes, mediated through MGBA. Certain strains can influence stress responses and interact with neurochemical pathways involved in mood regulation, sleep architecture, and cognitive function, highlighting their emerging relevance as psychobiotic agents.

Despite these promising findings, several limitations remain. Current studies are often characterized by methodological variability, and robust long-term clinical data in human populations are still limited. To address these gaps, rigorously designed clinical trials, combined with advanced approaches such as multi-omics technologies, are required to better elucidate underlying mechanisms and confirm therapeutic efficacy.

In conclusion, the integration of LAB into dietary strategies—whether through functional foods or targeted interventions—offers a practical and non-invasive approach to supporting both metabolic and mental health. Emerging evidence suggests that LAB may play a key role in future preventive and personalized healthcare strategies aimed at improving overall human health.

## Figures and Tables

**Table 1:** Recent literature compiling postbiotics: SCFAs and bacteriocins produced by LAB.

Postbiotic Class	Specific Metabolite	Producing Species/Strains	Reference
SCFAs	Acetic acid (Acetate)	*L. plantarum* *L. acidophilus* *L. casei* *L. rhamnosus* *B. animalis* subsp. *lactis*	[66]
	Propionic acid (Propionate)	*L. plantarum* *L. rhamnosus* *L. fermentum* *L. acidophilus* *B. longum* *B. bifidum*	[73,74,88]
	Butyric acid (Butyrate)	*L. acidophilus* *L. paracasei* *L. rhamnosus* *L. plantarum*	[73,89]
	Isovaleric/Isobutyric Acid	*L. plantarum* *L. paraplantarum* *L. fermentum* *L. acidophilus*	[74,90]
	Valeric acid	*L. acidophilus* *L. paraplantarum*	[74,90]
	Lactic acid	*Lactobacillus* spp. *B. animalis*	[66]
Bacteriocins	Bifidococcin	*B. longum* subsp. *infantis* LH_664	[86]
	Bifidin I	*B. longum* subsp. *infantis* BCRC 14,602 (partially sequenced)	[86,87]
	Bifidocin B	*B. bifidum* NCFB 1454	[86,87]
	Bisin	*B. longum* subsp. *longum* DJO10A	[86,87]
	Pentocin	*L. pentosus* ZFM94	[84]
	Helveticin 34.9	*L. helveticus* 34.9	[85]
	Bacteriocin ZFM54	*L. paracasei* ZFM54	[82]
	Bacteriocin-like inhibitory substances (BLIS)	*L. brevis* C23	[91]
	Bacteriocin 1.0320	*L. rhamnosus* 1.0320	[83]

**Table 2:** Key Mechanisms of the Gut-Microbiota-Brain Axis.

Pathway	Primary Mediators	Impact on Host Physiology	Reference
Neural	Vagus Nerve, GABA, Serotonin, Ach, Dopamine	Regulates mood, digestion, and blood pressure.	[169,170]
Endocrine	HPA Axis, Cortisol, Adrenocorticotropic Hormone (ACTH)	Mediates the stress response and gut permeability.	[172,176]
Immune	IL-6, TNF-α, IL-10, IL-12	Modulates systemic inflammation and neuroinflammation.	[138,139]
Metabolic	SCFAs (Butyrate, Acetate), BDNF	Supports MGBA integrity	[70]

**Table 3:** Different types of probiotic foods: microbial strains used and viability under various storage conditions.

Product Category	Food Matrix	Type of Food	Probiotic Strains	Relevant Added Ingredients	Time and Temperature of Storage	Viability After Storage	Reference
Dairy Food and Beverage	Milk	Fermented skim milk	*L. bulgaricus*, *L. acidophilus*, *L. rhamnosus*, *B. lactis*	Inulin	7 days at 4 °C	10^7^–10^9^ CFU/mL	[227]
		Fermented milk	*L. acidophilus* La-5	Mango juice (10%)	35 days at 4 °C	10^7^–10^8^ CFU/mL	[228]
		Yogurt	*B. bifidum*	Ginger extract and gum arabic	30 days at 4 °C	10^7^ CFU/mL	[229]
			*B. lactis*	Water-soluble soybean extract	29 days at 4 °C	10^6^–10^7^ CFU/mL	[230]
			*L. casei* 30-1	Lactitol (LAC) 15 g/L + microencapsulation (alginate + chitosan)	21 days at 4 °C	10^10^ CFU/g	[231]
			*L. acidophilus*, *B. animalis* subsp. *lactis*	Green banana pulp	45 days at 5 °C	10^6^–10^8^ CFU/g	[232]
	Cheese	Ricotta cheese	*B. animalis* subsp. *lactis* (Bb-12), *L. acidophilus* (La-05)	-	7 days at 7 °C	10^6^ CFU/g	[233]
		Cheddar cheese	*L. rhamnosus*	Nisin (0.05%)	28 days at 16 °C	10^8^ CFU/g	[234]
		Cottage cheese	*L. delbrueckii* UFV H2b20	Inulin (5%)	20 days at 5 °C	10^8^ CFU/g	[235]
		Semi-hard cheese	*L. paracasei* INIA P272	FOS (1%)	28 days at 12 °C	10^9^ CFU/g	[236]
		Scamorza cheese	*L. paracasei* subsp. *paracasei* IMC502, *L. rhamnosus* IMC501	-	30 days at 15 °C	10^9^ CFU/100 g	[237]
	Ice cream	Vanilla and fruit	*L. casei* DSM 20011, *L. rhamnosus* DSM 20021	Inulin (2.5–10%)	12 days at −20 °C	10^6^–10^7^ CFU/g	[238]
		Chocolate	*L. acidophilus* LA-5	Inulin (6.7%)	−18 °C	10^9^ CFU/100g	[239]
Plant-based food and beverages	Cereal-Based	African Maize-based fermented food	*L. rhamnosus* yoba 2012, *S. thermophilus* C106	-	28 days at 4 °C	10^7^–10^8^ CFU/g	[240]
		Pan bread	*L. acidophilus* LA-5, *L. casei* 431	Microencapsulation (alginate + Hi-maize resistant starch + chitosan coating); Inulin 5%	4 days at room temperature	>10^6^ CFU/g	[241]
		Bread	*L. plantarum* P8	-	5 days at 25 °C	10^6^–10^8^ CFU/g	[242]
		Riceberry malt beverage	*L. plantarum* TC24	Inulin (1.5%)	15 days at 8 °C	10^9^ CFU/mL	[243]
		Spice- and strawberry-flavoured non-dairy oats milk	Microencapsulated *L. plantarum*	Inulin	14 days at 4 °C	>10^8^ CFU/mL	[244]
		Oat-based beverage	*L. plantarum*	Inulin (1%), pectin (0.4%), λ-carrageenan (0.2%), vitamin C (0.06%), citric acid (0.15%), minerals and vitamin E	49 days at 4 °C	>10^7^ CFU/g	[245]
		Fruit-flavoured rice milk	*L. acidophilus*, *B. bifidum* BB-1, *S. thermophilus*	Mango or papaya pulp (10–20%)	15 days at 4 °C	>10^8^ CFU/mL	[246]
		Fermented rice milk with cactus pear and physalis pulp	*L. acidophilus* *B. bifidum* BB-1 *S. thermophilus*	Cactus pear or physalis pulp	15 days at 4 °C	>10^7^ CFU/mL	[247]
		Rice milk	*L. casei*, *B. longum*, *L. bulgaricus*, *S. thermophilus*, *L. acidophilus*	Honey 5%	21 days at 5 °C	>10^6^ CFU/mL	[248]
		Riceberry/sesame–riceberry milk ice cream	*L. casei* 01, *L. acidophilus* LA5	Inulin	60 days at −25 °C	10^6^–10^7^ CFU/g	[249]
	Pseudo-cereal-based	Quinoa beverage	*Lb. plantarum* DSM 9843	-	28 days at 4 °C	~−10^7^ CFU/mL	[250]
		Quinoa beverage	*Bifidobacterium* spp., *L. acidophilus*, *S. thermophilus*	Raspberry syrup 10%	21 days at 5 °C	>10^6^ CFU/mL	[251]
		Fermented buckwheat	*L. rhamnosus* GG	Chocolate or caramel	14 days at 6 °C	10^8^–10^9^ CFU/mL	[252]
	Legume-Based	Soyabean milk (cow milk/camel milk + soyabean extract)	*L. acidophilus* LA-5, *B. animalis* subsp. *lactis* Bb-12, *L. casei* LC-01, *S. thermophilus* Th-4, *L. delbrueckii* ssp. *bulgaricus*	-	21 days at 4 °C	10^6^–10^7^ CFU/mL	[253]
		Chickpea and coconut beverage	*L. paracasei* subsp. *paracasei* LBC 81	-	10 days at 4 °C	>10^8^ CFU/mL	[254]
		Soyabean/peanut based mixed beverage	*Lb. rhamnosus* GG	-	42 days at 8 °C	>10^7^ CFU/mL	[255]
		Soymilk	*L. acidophilus* La-5, *B. animalis* subsp. *lactis* Bb-12, *S. thermophilus*	FOS and/or inulin (40 g/kg)	28 days at 5 °C	~10^6^–10^8^ CFU/mL	[256]
			*L. casei* PLA5	FOS, inulin, mannitol (10 g/L)	10 days at 4 °C	>10^7^ CFU/mL	[257]
		Soymilk yogurt	*B. animalis* subsp. *lactis* BB-12, *L. acidophilus* La-5, *L. rhamnosus*	-	28–30 days at 4 °C	>10^8^ CFU/mL	[258,259]
		Peanut-based cheese substitute	*L. rhamnosus* NCDC18	-	15 days at 4 °C	10^6^ CFU/mL	[260]
		Fermented roasted peanut milk	*B. longum* BB536	Arabic gum 10% (*v*/*v*)	14 days at 5–7 °C	>10^6^ CFU/mL	[261]
	Fruit Juice	Cornelian cherry juice	*L. casei* T4 or *L. plantarum* ATCC 14917	-	28 days at 4 °C	10^8^–10^9^ CFU/mL	[262,263]
		Apple juice	*L. paracasei* ssp. *paracasei* (LC-01)	Oligofructose (20 g/L)	28 days at 4 °C	10^6^–10^7^ CFU/mL	[264]
		Pineapple juice	*L. plantarum* 299V, *L. acidophilus* La5, *B. lactis* Bb-12	Oligofructose (1%)	60 days at 4 °C	10^8^–10^10^ CFU/mL	[265]
		Orange juice	*P. acidilactici* CE51	-	35 days at 4–30 °C	10^7^–10^8^ CFU/mL	[266]
		Pomegranate juice	*L. plantarum* ATCC 14917	-	28 days at 4 °C	>10^8^ CFU/mL	[267]
	Vegetable-based juices	Yacon, beetroot, tomato, carrot, cabbage, cucumber, pumpkin, turnip	*L. acidophilus*, *L. plantarum*, *L. casei*, *L. rhamnosus*, *L. delbrueckii*, *B. lactis*	Inulin, FOS or microencapsulation	From 15–28 days to 35–56 days at 4 °C	10^6^–10^9^ CFU/mL	[268,269]
	Nut-Based	Cashew nut milk	*B. animalis* BB-12	-	30 days at 4 °C	>10^8^ CFU/mL	[270]
		Functional kefir (hazelnut-milk (75%) and cow milk (25%))	*Lactobacillus* spp., *Lactococcus* spp.	Yeast (kefir starter culture)	21 days at 4 °C	>10^7^ CFU/mL	[271]
		Almond milk powder	*L. plantarum*	Maltodextrin 6% (*w*/*v*)	240 days at 4 °C	>10^7^ CFU/g	[272]
		Almond yogurt	*S. thermophilus*, *L. delbrueckii* subsp. *bulgaricus*, *L. acidophilus*, *B. lactis*	-	21 days at 4 °C	>10^7^ CFU/g	[273]
Edible coatings	Baked food	Baked cereal products	*L. acidophilus*, *L. rhamnosus* GG, *B. animalis* BB-12	Edible films (alginates, whey protein, methylcellulose, starches, glycerol) (applied to the surface after cooking)	1–90 days at 4 °C	10^6^–10^7^ CFU/g (bread crust)	[274]
		Pan bread (crust)	*L. rhamnosus* GG	Sodium alginate (ALG) + Whey Protein Concentrate (WPC) + glycerol (applied after cooking)	7 days at 25 °C	10^7^–10^9^ CFU/portion	[275]
	Fruit	Strawberries	*L. plantarum* PTCC 1058	Carboxymethylcellulose (CMC) + glycerol (30% *w*/*w*)	16 days at 4 °C	>10^6^ CFU/g	[276]
		Blueberries	*L. rhamnosus* CECT 8361	Alginate + Glycerol + Inulin + Oligofructose	21 days at 5 °C	>10^6^ CFU/g	[277]

**Table 4:** Clinical evidence on the effects of various probiotic microorganisms on mental health.

Food or Probiotic Supplementation	LAB and Bifidobacteria Strain	Study Model	Patients Model and Pathology	Weeks of Treatment	Mental-Health Outcomes	Reference
Fermented milk	*L. casei* strain Shirota YIT 9029 (10^9^–10^11^ CFU/100 mL)	Double-blind, placebo-controlled, parallel-group study (DBPC-parallel group study)	Medical students under academic stress (*n* = 47)	8 weeks	↓ Stress-associated abdominal dysfunction; ↑ microbiota diversity, salivary cortisol levels	[288,289]
Fermented milk	*L. casei* strain Shirota YIT 9029 (10^9^ CFU/100 mL)	Double-blind, placebo-controlled, parallel-group study (DBPC-parallel group study)	Medical students under academic stress (*n* = 94)	11 weeks	↓ Sleep disturbance	[290]
Yogurt	*L. delbrueckii* ssp. *bulgaricus* OLL1073R-1, *S. thermophilus* (≥1.12 × 10^9^ CFU/mL)	Randomized controlled trial study (RCT)	Women healthcare workers (*n* = 479)	16 weeks	↑ Psychological quality of life, sleep quality, vitality	[291]
Tempeh-derived probiotic preparation	*L. fermentum* A2.8 (10^8^ CFU/mL)	Experimental intervention study (Exp. Int. Study)	Adult elderly with cognitive impairment (*n* = 93)	12 weeks	↑ Cognitive functions (memory, learning process, verbal fluency, visuospatial function)	[292]
Fermented soybean capsules (DW2009)	*L. plantarum* C29 (1.25 × 10^10^ CFU/g)	Multi-center, double-blind, randomized, placebo-controlled study (Multicenter, DB-RCT)	Adults diagnosed with MCI (*n* = 100)	12 weeks	↑ Cognitive functions (memory and attention), BDNF	[293]
LAB tablets	*L. helveticus* MIKI-020 (0.2 g)	Double-blind, randomized, placebo-controlled, crossover study (DB-RPCC)	Adult patients with sleep disorders (*n* = 40)	4 weeks	↑ Sleep efficiency, motivation, calmness	[294]
LAB capsules	LAB mix (0.33 g/capsule)	Randomized controlled clinical study (RCT)	Adult patients with SIBO, depression and diabetes (*n* = 60)	1 week	↓ Anxiety, depression (SAS-SDS) scores, IL-2, TNF-α	[295]
Probiotic fortified kefir	*L. helveticus* R0052, *B. longum* R0175 (6 × 10^9^ CFU/mL)	Double-blind, randomized, placebo-controlled study (DBRPCT)	Elderly men with depression (*n* = 67)	8 weeks	↑ Appetite, TAC ↓ GDS-15 score	[296]
Capsules	*L. acidophilus*, *L. casei*, *B. bifidum* (2 × 10^9^ CFU/g)	Double-blind, randomized, placebo-controlled study (DBRPCT)	Adult patients with MDD (*n* = 40)	8 weeks	↑↑ Depressive symptoms measured by Beck ↓↓ Depression Inventory (BDI)	[297]
Capsules	*L. reuteri* NK33, *B. adolescentis* NK98	Double-blind, place-bo-controlled, parallel-group study (DBPC-parallel group study)	Healthy adults patients with psychological stress and subclinical symptoms of depression, anxiety and insomnia (*n* = 156)	8 weeks	↓↓ depressive and anxiety symptoms ↑ sleep quality and insomnia symptoms	[298]
Capsules	*L. reuteri* NK33, *B. adolescentis* NK98	Double-blind, randomized, placebo-controlled study (DBRPCT)	Adult patients with subclinical symptoms of depression, anxiety, and insomnia (*n* = 156)	8 weeks	↓ Anxiety/depression; ↑ sleep quality (especially sleep induction)	[292]
Sachets	*B. breve* A-1 (10^11^ CFU/day)	Open-label, single-arm study	Adult outpatients with schizophrenia and anxiety-depressive symptoms (*n* = 29)	4 weeks	↑↑ Anxiety/depression	[299]
Sachets	*B. longum* NCC3001 (10^10^ CFU/day)	Double-blind, randomized, placebo-controlled study (DBRPCT)	Adult patients with IBS (diarrhoea/mixed) and mild to moderate anxiety or depression (*n* = 44)	6 weeks	↑↑ Depression ↑ physical quality of life	[300]
Yogurt	*Bifidobacterium* spp.	Interventional study (pre-post, non-placebo-controlled)	Adult patients with low mood/poor well-being (*n* = 98)	8 weeks	↓↓ Depression and psychological well-being	[301]
Yogurt	*B. animalis* ssp. *lactis* BB-12 (1 × 10^9^ CFU/100 g)	Randomized controlled trial study (RCT)	Female taekwondo athletes (*n* = 51)	8 weeks	↑ Psychological fatigue, cognitive and emotional function	[302]
Fermented milk	*B. bifidum* YIT 10347	Open-label, single-arm pilot study	FGID patients (*n* = 37)	4 weeks	↓ salivary stress markers ↑↑ TMD/POMS	[303]
Yogurt	*Lactobacillus gasseri* SBT2055, *B. longum* SBT2928 (100 g/day)	Double-blind, randomized, placebo-controlled study (DBRPCT)	Healthy adult volunteers (*n* = 224)	12 weeks	↑ Natural Killer cell activity ↓ Serum ACTH levels	[304]
Capsules	*L. rhamnosus* and *B. lactis* (3.3 × 10^9^ CFU/capsule)	Double-blind, randomized, placebo-controlled, crossover study (DBRPCC)	Healthy adults (*n* = 33)	10 weeks	↑ Orientation, concentration/ calculation, and memory (MMSE) ↑ verbal working and visuo-spatial working memory; ↑ BDI, STAI, Sleep quality (PSQI)	[305]
Sachets	*B. breve* CCFM1025 (10^10^ CFU/day)	Double-blind, randomized, placebo-controlled study (DBRPCT)	Adult MDD patients (*n* = 45)	4 weeks	↓ Depression	[306]

Abbreviation: ↑, increased; ↓, decreased; MCI, Mild Cognitive Impairment; SIBO, Small Intestinal Bacterial Overgrowth; SAS, Self-Rating Anxiety Scale; SDS, Self-Rating Depression Scale; TAC, Total Antioxidant Capacity; GDS-15, Geriatric Depression Scale 15; TMD, Total Mood Disturbance; POMS, Profile of Mood States; MMSE, Mini-Mental State Examination; BDI, Beck Depression Inventory; STAI, State-Trait Anxiety Inventory; PSQI, Pittsburgh Sleep Quality Index.

## Data Availability

No new data were created or analyzed in this study. Data sharing is not applicable to this article.

## Abbreviations

The following abbreviations are used in this manuscript:

ACE	Angiotensin-Converting Enzyme
ACH	Acetylcholine
ACTH	Adrenocorticotropic Hormone
BDNF	Brain-Derived Neurotrophic Factor
BSH	Bile Salt Hydrolase
CNS	Central Nervous System
CVD	Cardiovascular Disease
DCs	Dendritic Cells
EPS	Exopolysaccharides
ENS	Enteric Nervous System
FDA	US Food and Drug Administration
FFAR2	Free Fatty Acid Receptor 2
FFAR3	Free Fatty Acid Receptor 3
GI	Glycemic Index
GLP-1	Glucagon-Like Peptide-1
GSH-Px	Glutathione Peroxidase
GRAS	Generally Regarded As Safe
HDL	High-Density Lipoprotein
HMG-CoA	3-Hydroxy-3-Methylglutaryl-Coenzyme A
HPA	Hypothalamic–Pituitary–Adrenal Axis
IL	Interleukin
ISAPP	International Scientific Association for Probiotics and Prebiotics
LAB	Lactic acid bacteria
LDL	Low-Density Lipoprotein
LPS	Lipopolysaccharide
LTA	Lipoteichoic Acid
MDD	Major Depressive Disorder
MGBA	Microbiota–Gut–Brain Axis
MUBs	Mucus-Binding Proteins
NCD	Non-Communicable Disease
NF-κB	Nuclear Factor Kappa-B
NK	Natural Killer
QPS	Qualified Presumption of Safety
PPAR	Peroxisome Proliferator-Activated Receptor
PRRs	Pattern Recognition Receptors
SLP	Surface Layer Protein
TGF-β	Transforming Growth Factor Beta
Th	T-Helper Cells
TLR	Toll-Like Receptor
TNF-α	Tumor Necrosis Factor-alpha
USP	United States Pharmacopeia
